# Availability, cost and affordability of essential medicines for smoking cessation in low-income and middle-income countries: a cross-sectional study

**DOI:** 10.1136/thorax-2024-222391

**Published:** 2025-02-03

**Authors:** Catherine Plum, Marie Stolbrink, Obianuju Ozoh, Shamanthi Jayasooriya, Rebecca Nightingale, Kevin Mortimer, David Halpin

**Affiliations:** 1 University Hospitals of Morecambe Bay NHS Trust, Lancaster, UK; 2 Department of Clinical Sciences, Liverpool School of Tropical Medicine, Liverpool, UK; 3 Department of Pulmonology, Stellenbosch University, Stellenbosch, South Africa; 4 Department of Medicine, University of Lagos College of Medicine, Lagos, Nigeria; 5 Pulmonology, Lagos University Teaching Hospital, Surulere, Lagos, Nigeria; 6 Academic Unit of Primary Care, Medical Research Council The Gambia, Sheffield, UK; 7 Liverpool School of Tropical Medicine, Liverpool, UK; 8 Department of Pathology, University of Cambridge, Cambridge, Cambridgeshire, UK; 9 Department of Paediatrics and Child Health, University of KwaZulu-Natal, Durban, South Africa; 10 Respiratory Medicine, Liverpool University Hospitals NHS Foundation Trust, Liverpool, UK; 11 Department of medicine, University of Exeter Medical School, Exeter, UK

**Keywords:** Smoking cessation, Tobacco control

## Abstract

Smoking cessation is more effective when supported by medicines. Data on the availability, cost and affordability of these treatments in low-income and middle-income countries (LMIC) are limited. Cross-sectional data for smoking cessation medications were collected from pharmacies, healthcare facilities and central medicine stores in 60 LMIC (2022–2023). Medications had varying availability, large price ranges and were essentially unaffordable. Enabling access to these medications is important in reducing tobacco consumption and associated disease. Strategies for integrating smoking cessation services into health systems are needed to reach Sustainable Development Goal targets.

## Introduction

 The WHO Sustainable Development Goals (SDG) target a reduction in tobacco consumption by 30% by 2025, as part of the strategy to reduce premature deaths from non-communicable disease by a third by 2030.^1^ The WHO MPOWER measures outline ways to reduce tobacco consumption, including ‘offering help to quit’.^2^ Over 80% of tobacco smokers live in low-income and middle-income countries (LMIC),^3^ and smoking cessation medications should be accessible and affordable to all.^4^

Smoking cessation medications including nicotine gums, transdermal patches, bupropion and varenicline tablets have been on the WHO ‘Model List of Essential Medicines’ (EML) since 2009, based on their efficacy, cost-effectiveness and safety.^5^ Evidence on the availability, cost and affordability of these treatments in LMIC is limited. This study provides high-quality data describing the current accessibility of these medications in LMIC.

## Methods

Healthcare professionals in LMIC completed a survey on the availability and cost of smoking cessation medications from three facility types: a pharmacy, healthcare facility (HCF) and central medicine store (CMS, online supplemental appendix). Collaborators were recruited through professional networks and snowballing. Supply for 1 month’s treatment was based on average cigarette consumption of 10/day.^6^ Prices for 1 month’s treatment were compared. A medicine was considered affordable if 1 month’s treatment cost less than 1 day’s wage of the lowest paid government worker, defined by local minimum wage.^7^ Global varenicline supply was interrupted in 2021, so it was not explored in this study.^8^ This survey was nested in the *Thorax*-published study on the accessibility of chronic respiratory disease medicines (online supplemental appendix).^9^

## Results

Data were reported from 60 countries, representing 84% of the global LMIC population. Information from pharmacies, HCFs and CMSs was available from 57, 56 and 46 countries, respectively. Most pharmacies were private (51/60), and more HCFs were public (45/60). There were 18 low-income (LIC), 24 low-middle-income, 17 upper-middle-income (UMIC) countries; Venezuela was unclassified (online supplemental appendix). Africa was the best-represented region with 26/60 countries.

### Nicotine gum

**Figure 1:**
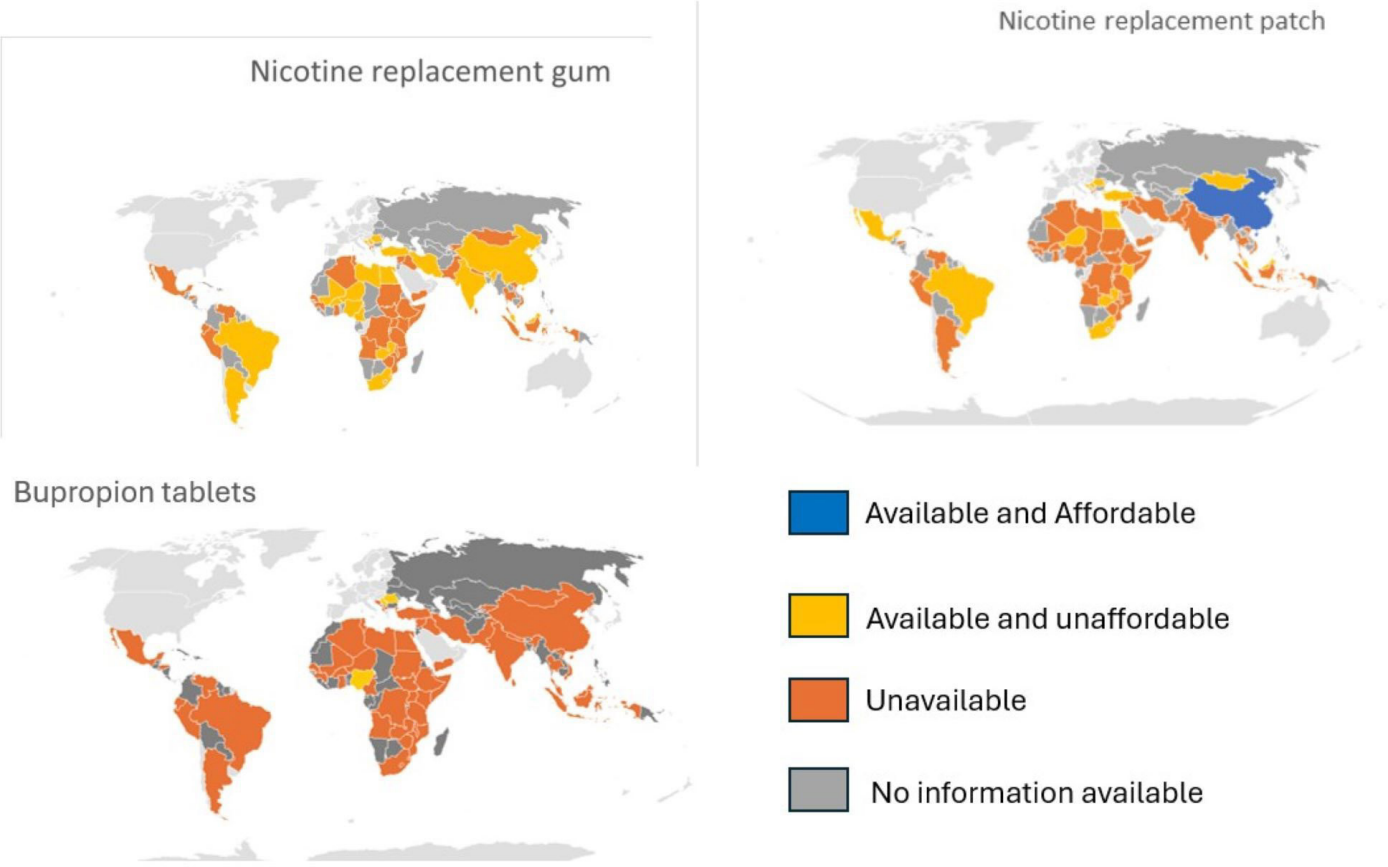
Availability and affordability of nicotine gum, nicotine patches and bupropion by region.

Nicotine gum was available in 20/57 pharmacies, 5/56 HCFs and 4/46 CMSs. Doses ranged from 2 to 4 mg(table 1, figure 1, online supplemental appendix). Price was provided by 19/20 pharmacies, 5/5 HCFs and 2/4 CMSs. The median (IQR) cost was US$96 (US$65.13–123.98), US$61.94 (US$55.88–89.05) and US$65.72 (US$11.44–120.00) in pharmacies, HCFs and CMSs, respectively. It was unaffordable from all facilities that provided price information (figure 1, online supplemental appendix).

**Table 1:** Comparisons of availability, cost and affordability of WHO EML medicines for smoking cessation in LMIC

Facility	Survey measurement	Smoking cessation medications		
		Nicotine gum(2 mg, 4 mg)	Nicotine transdermal patch (15–30 mg/16 hours; 7–21 mg/24 hours)	Bupropion tablets (150 mg)
**Pharmacy**	Availability	20/57	13/57	2/57
	Median cost (IQR, US$)	96(65.13–123.98)	62.09(51.54–100.90)	143.89(129.38–158.40)*
	Median DOW (IQR)	10.35(5.74–51.07)	5.84(3.16–40.03)	31.76(6.37–57.16)*
**HCF**	Availability	5/56	6/56	2/56
	Median cost (IQR, US$)	61.94(55.88–89.05)	68.61(43.80–307.31)	25.10(23.42–26.78)*
	Median DOW (IQR)	3.81(3.26–7.70)	27.78(3.01–60.53)	1.38(1.32–1.43)*
**CMS**	Availability	4/46	5/46	1/46
	Median cost (IQR, US$)	65.72(11.44–120.00)	43.72(12.00–124.29)	NIA

Cost: Supply for one month's treatment was guided by average daily cigarette consumption in LMIC of 10 cigarettes/day. One month's supply of nicotine gums was 10 gums/day, 30 transdermal patches (1 patch/day) and for bupropion 60 tablets/month based on average maintenance dose. Affordability was not calculated for CMSs as their prices were wholesale prices.

* IQR could not be calculated due to missing information on price so results given as median (minimum-maximum).

CMS, central medicine store; DOW, days of work required to pay for 1 month's treatment; HCF, healthcare facility; LMIC, low-income and middle-income countries; NIA, no information available.

### Nicotine patch

Nicotine transdermal patches were available in 13/57 pharmacies, 6/56 HCFs and 5/46 CMSs (table 1, online supplemental appendix). Doses ranged from 1 to 21 mg. Price was provided by 13 pharmacies, five HCFs and three CMSs. The median (IQR) cost was US$62.09 (US$51.54–100.90), US$68.61 (US$43.80–307.31) and US$43.72 (US$12.00–124.29) in pharmacies, HCFs and CMSs, respectively. It was affordable in one pharmacy (China) (figure 1, online supplemental appendix).

### Bupropion tablets

Bupropion 150 mg tablets were available in 2/57 pharmacies, 2/56 HCFs and 2/46 CMSs. Two pharmacies and two HCFs gave price information. The median (IQR) cost of tablets was US$143.89 (US$–151.15) and US$25.10 (US$24.26–25.94) in pharmacies and HCFs, respectively. Bupropion was unaffordable from all facilities that provided price information (table 1, figure 1, online supplemental appendix).

## Discussion

This survey provides up-to-date, high-quality data on the availability, cost and affordability of smoking cessation medications globally and is the largest cross-sectional study on this topic in LMIC to date. Data came from a variety of income levels and 16 out of 20 of the most populous LMIC.

WHO EML medications for smoking cessation were not widely available and were largely unaffordable for those living in LMIC. The most available form was nicotine gum, and the least available was bupropion. All pharmacotherapies were more available in UMIC (online supplemental appendix, table 2). Our data showed wide variability in price.

**Table 2:** Availability of WHO EML medications for smoking cessation by World Bank country income level

Medication	Availability	Low-income country (n=18)	Low-middle-income country (n=24)	Upper-middle-income country (n=17)
Nicotine gum(2 mg, 4 mg)	Pharmacy	5	5	10
	HCF	0	1	4
	CMS	0	1	3
Nicotine transdermal patch(15–30 mg/16 hours; 7–21 mg/24 hours)	Pharmacy	3	2	8
	HCF	0	3	4
	CMS	0	2	3
Bupropion tablets(150 mg)	Pharmacy	0	1	1
	HCF	0	0	2
	CMS	0	0	2

Pharmacies n=57 LMIC, HCF n=56 LMIC, CMS n=46 LMIC. Venezuela was not classified.

CMS, central medicine store; HCF, healthcare facility; LMIC, low-income and middle-income countries.

Medicines were more available in pharmacies but more expensive (table 1), which is likely to be an accessibility barrier.

Results showed that these medications were mostly unaffordable, especially as treatment typically lasts at least 3 months. From our data, the median cost for 1 month of cigarettes is US$12.27(IQR US$7.71–21.23) (online supplemental appendix), cheaper than the cost of any medication to support smoking cessation (table 1). The latest WHO report suggests that LMIC needs to raise taxes and prices of cigarettes.^10^ Our data show the importance of coordinated implementation of the MPOWER measures as ‘offering to help quit’ is economically undermined if cigarettes remain more affordable than treatment.

The strengths of the study were the wide range of included LMIC and the actual determination of availability and cost by health professionals. There were some limitations including sampling bias towards urban, large centres. Participants were asked to enter the cheapest price of gum, patch and tablet per facility, so there could be more price variety and higher median costs. Information on prices of tablets from HCFs and CMSs was limited and some facilities had missing data. Since data collection, there has been an update to the WHO EML in July 2023, which now includes other forms of nicotine replacement therapy; lozenges and oral spray.

## Conclusion

Everyone globally should have the best support in stopping smoking, but without medications chances of successful smoking cessation are reduced. Our data can be used to inform smoking cessation programmes in LMIC, and combining this with other strategies will be key in achieving the greatest success for the individuals, as well as global targets in disease reduction.

## Supplementary material

10.1136/thorax-2024-222391 online supplemental file 1
