# Supplementary material for: Availability, cost and affordability of essential medicines for smoking cessation in low-income and middle-income countries: a cross-sectional study
Source: Thorax. 2025 Feb 3;80(4):e222391. doi: 10.1136/thorax-2024-222391 (PMC12015041; doi:10.1136/thorax-2024-222391)
Supplement: online supplemental file 1 [file thorax-80-4-s001.pdf]

Appendix: Availability and affordability of medications for smoking cessation in LMIC

Table of Contents page 1

- Full methods….Page 2-3
- Figure 1: Flowchart of sampling strategy for potential collaborators….page 3
- Supplementary Table 1: Medicines to treat tobacco addiction on the WHO EML (2021)....page 4
- Supplementary table 2: Chronic respiratory diseases medicines survey investigators….page 5-9
- Supplementary Table 3: Strobe statement….page 10-13
- Supplementary Table 4: Overview of included LMICs and facilities by income group and WHO region….page 14-16
- Supplementary table 5: Availability, Cost and affordability of Nicotine gums in pharmacies, HCFs and CMSs in LMIC….page 17-20
- Supplementary Table 6: Availability, cost and affordability of nicotine transdermal patches in pharmacies, HCFs and CMSs in LMIC….page 21-24
- Supplementary Table 7: Availability, cost and affordability of Bupropion or Varenicline tablets in pharmacies, HCFs and CMSs in LMIC….page 25-28
- Supplementary table 8: Availability of medications for smoking cessation by WHO regions and income level in pharmacies, HCFs and CMSs… page 29-35
- Supplementary Table 9: Cost of cigarettes in LMIC: One month's cost based on average consumption of 10 cigarettes/day…. Page 36-38
- Varenicline and Bupropion limitations … Page 39
- Supplementary table 10: Data collected before and after Bupropion supply disruption 1/12/2022… page 39
- References…. page 40-41

Full methods

Study Design

This was a cross sectional survey completed by healthcare professionals working in LMIC between June 2022 and April 2023, on the availability and cost of medications for smoking cessation. This study was nested in a larger cross-sectional survey exploring medicines for chronic respiratory diseases in LMIC, and therefore study design, methods and data collection is the same (1). Ethical approval was granted from the Liverpool School of Tropical Medicine (LSTM), and the study followed STROBE guidelines (2) (Supplementary Table 3). Each collaborator completed a form on the kobotoolbox platform which collected standardised electronic data on demographics, availability and prices of medicines (3). Local approval, if necessary, and informed consent were gained. Essential medicines were defined by the WHO EML 2021 (4)(Table 1). Availability, strength, pack size and price were recorded for each medicine. Data for cigarette prices was from the Global Health Observatory data repository (5). Each collaborator was asked to complete one form for three facilities: one pharmacy, one healthcare facility (HCF, for example clinic or hospital) and one central medicine store (CMS), including public and private facilities. Multiple entries for each country and facility were allowed. If facilities were missing the authors contacted other collaborators to complete the information or used publicly available data to complete. Participants were encouraged to enter the cheapest product and price that the patient would pay.

Sampling and recruitment

Participants were recruited through international networks and through personal connections ("snowballing") (Figure 1), in order to achieve the most representative sample possible. LMICs were defined by the 2022 World Bank category (6). There was no predefined sample size.

Figure 1: Flowchart of sampling strategy for potential collaborators

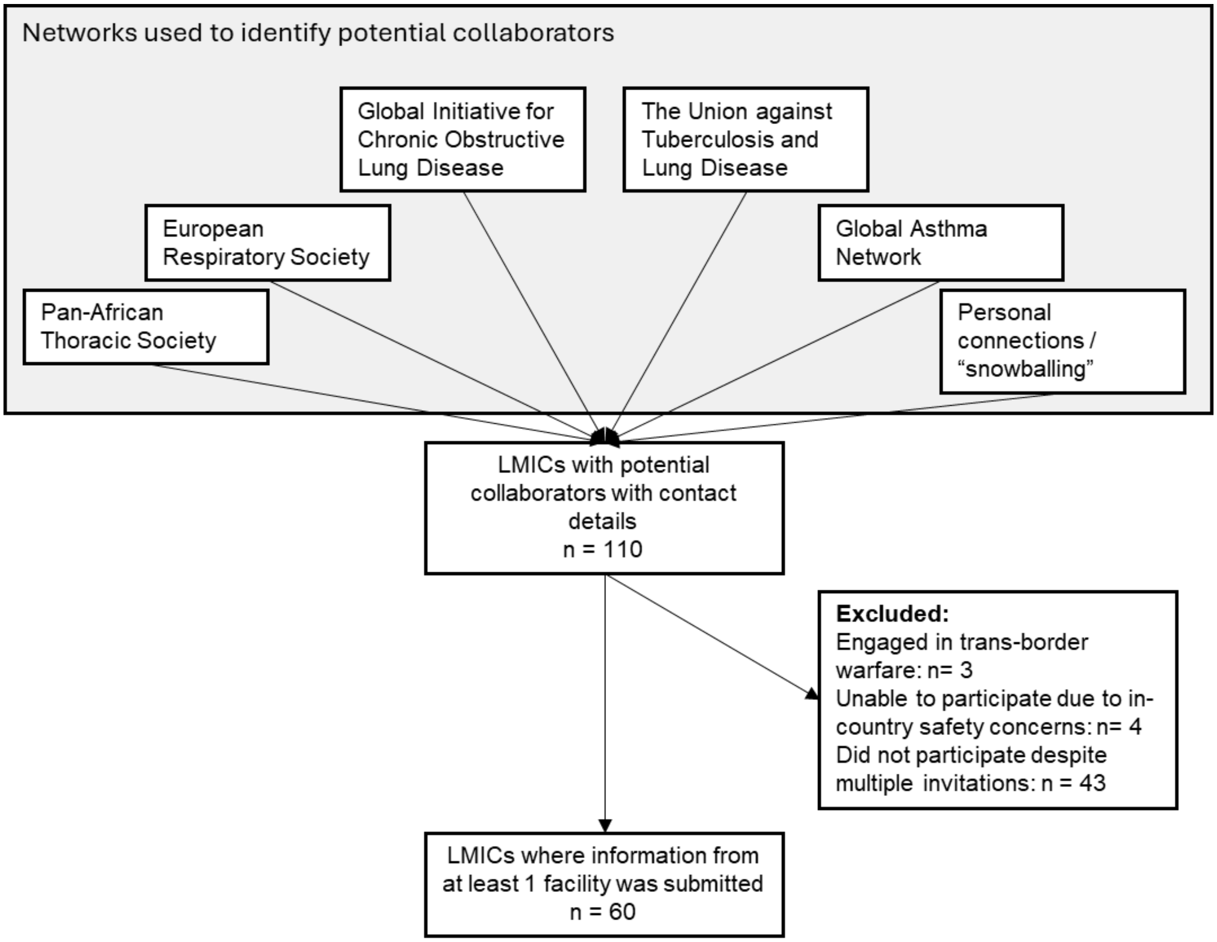

Data analysis

For each country the availability and cost of each medicine at the specified doses was reported for each of the three types of facility. Prices of medicines were initially expressed in local currency, then converted to US dollars using daily or mean monthly exchange rates for the month that the data was submitted [7]. Supply for one month's treatment was guided by average daily cigarette consumption in LMIC of 10 cigarettes/day [8] and treatment guidelines. Therefore one month's supply of nicotine gums was 10 gums/day for 30 days, 30 transdermal patches (1 patch/day for 30 days) and for Bupropion and Varenicline 60 tablets/month based on average maintenance dose for one month [9, 10]. If one month's treatment costs less than one day's wage of the lowest paid government worker, defined by national minimum wage [11], the medicine was considered affordable. This is as per established methodology [12, 13] Affordability was not calculated for the CMS as their prices were wholesale prices.

Supplementary Table 1: Medicines to treat tobacco addiction on the WHO EML (2021)(4)

Bupropion	Tablet sustained release: 150 mg (hydrochloride)
Nicotine replacement therapy	Chewing gum: 2mg, 4mg (as parocrilex)<br><br>Transdermal patch: 15mg to 30mg/16 hours; 7mg to 21mg/24 hours
Varenicline	Tablet: 0.5mg to 1 mg

Supplementary Table 2: Chronic Respiratory Disease Medicines Survey investigators (Collaborators and Institutions

Country	First name	Surname	Affiliation
Albania	Eris	Mesonjesi	University Hospital Center Mother Teresa, Tirana
Algeria	Nadia	Ait-Khaled	Algeria University
Algeria	Samya	Taright	Universite d'Alger, Faculté de Medecine
Argentina	Santiago	Larrateguy	Universidad Adventista del Plata / Centro Privado de Medicina Respiratoria
Bosnia and Herzegovina	Sanela	Domuz Vujnovic	Paediatrics Clinic, University Clinical Center of Republic of Srpska
Brazil	Carolina	Barbosa Souza Santos	Programa para o Controle da Asma na Bahia - ProAR (Program for Control of Asthma in Bahia)
Burkina Faso	Abdoul Risgou	Ouédraogo	Health Training & Research Unit, Joseph KI-ZERBO University, Ouagadougou, Burkina Faso and Department of Medicine, Tengandogo University Hospital Center
Cameroon	Bertrand Hugo	Mbatchou-Ngahane	Douala General Hospital, University of Douala
Cameroon	Lydie	Mboumi	Pharmacie du Rocher, Douala
China	Yanping	Liu	Sun Yat-sen university
China	Fu-Qiang	Wen	West China Hospital, Sichuan University
China	Xi	Yan	West China Hospital, Sichuan University
China	Yutian	Zhang	West China Hospital, Sichuan University
Congo, Dem. Rep.	Patrick	Katoto	Center for Tropical Diseases and Global Health, Université Catholique de Bukavu
Congo, Dem. Rep.	Arsene Daniel	Nyalundja	Center for Tropical Diseases and Global Health, Université Catholique de Bukavu
Ecuador	Efraín	Sánchez-Angarita	Centro de Investigacion respiratorio
Egypt, Arab Rep.	Maged	Hassan	Alexandria University, Faculty of Medicine
Egypt, Arab Rep.	Magda	Afifi	National Tuberculosis Control Program, Ministry of health and population
Eswatini	Willie	Siduna	University of the Western Cape

Ethiopia	Amsalu	Binegdie	College of Health Sciences, Addis Ababa University
Gambia, The	Babatunde	Awokola	Liverpool School of Tropical Medicine and Medical Research Council Unit The Gambia at London School of Hygiene and Tropical Medicine
Ghana	Rafiuk Cosmos	Yakubu	Tamale Teaching Hospital (TTH) and School of Medicine, University for Development Studies (UDS)
Guinea	Magassouba	Aboubacar Sidiki	National Tuberculosis Control Program,Conakry
Honduras	Suyapa	Sosa	Pulmonary Medicine/Thorax National Institute
India	Sarbjeet	Khurana	IHBAS Hospital
Indonesia	Bony Wiem	Lestari	Research Center for Care and Control of Infectious Diseases (RC3ID) Universitas Padjadjaran, Bandung
Indonesia	Faisal	Yunus	Department of Pulmonology and Respiratory medicine, faculty of medicine universitas Indonesia - Persahabatan Hospital
Indonesia	Antonia Morita Iswari	Saktiawati	Universitas Gadjah Mada, Faculty of Medicine, Public Health, and Nursing, Department of Internal Medicine
			Universitas Gadjah Mada, Faculty of Medicine, Public Health, and Nursing, Center for Tropical Medicine
Iran, Islamic Rep.	Mohammad Reza	Masjedi	Pulmonary Medicine, Shahid Beheshti University of Medical Sciences
			Cancer Control Research Center, Cancer Control Foundation, Iran University of Medical Sciences, Tehran
			Tobacco Control Research Center (TCRC), Iranian Anti-tobacco Association, Iran University of Medical Sciences, Tehran
Iraq	Hashim	Talib Hashim	University of Warith Al-anbya, Colleges of Medicine, Karbala
Kenya	Peter	Owiti	Stop TB Partnership / Wote Youth Development Projects
Kyrgyz Republic	Shaiirbek	Sulaimanov	Kyrgyz-Russian Slavic University and Kyrgyz State Medical Academy
Lesotho	Lawrence	Oyewusi	Partners In Health
Libya	Boshra	Abusahmin	National Centre For Disease Control
Libya	Mohamed Hadi Mohamed	Abdelhamid	National Center for Disease Control (NCDC), Biotechnology Research Center (BTRC), Tripoli
Malawi	Felix	Mkandawire	Blantyre Malaria Project
Malaysia	Ee Ming	Khoo	Department of Primary Care Medicine, Faculty of Medicine, Universiti Malaya; International Primary Care Respiratory Group

Mali	Ousmane Ibrahim	Diabate	Bamako
Mexico	Adrian	Rendon	CIPTIR, Hospital Universitario "Dr. Jose Eleuterio Gonzalez", UANL
Mexico	Berenice	Soto-Moncivais	CIPTIR, Hospital Universitario “Dr. Jose Eleuterio Gonzalez”, UANL
Mongolia	Bolyskhan	Baigabyl	Tuberculosis clinic, National Center for Communicable Diseases
Mozambique	Celso	Khosa	Instituto Nacional de Saúde- Centro de Investigação e Treino em Saúde da Polana Caniço (CISPOC), Marracuene
Mozambique	Cynthia	Silva	Instituto Nacional de Saúde- Centro de Investigação e Treino em Saúde da Polana Caniço (CISPOC), Marracuene
Nepal	Rajan	Paudel	Karolinska Institutet
Niger	Alberto	Piubello	Damien Foundation, Niamey
Niger	Kadri	Sani	Centre Hospitalier Régional, Niamey
Nigeria	Temitope	Fapohunda	Lagos State University Teaching Hospital
Nigeria	Olayinka	Adeyeye	Lagos State University, College of Medicine
North Macedonia	Valentina	Cvejoska Cholakovska	University Children's Clinic, Faculty of Medicine, Skopje, Ss Cyril and Methodius University of Skopje
Pakistan	Ghulam	Mustafa	College of Medicine Shaqra University Riyadh, Nishtar medical university, Multan
Peru	Javier	Cabrera-Sanchez	Facultad de Medicina, Universidad Peruana Cayetano Heredia, Lima
Romania	Diana	Deleanu	University of Medicien & Pharmacy Iuliu hatieganu
Rwanda	Jean Pierre	Sibomana	University of Rwanda, King Faisal Hospital Kigali
Senegal	Momar	Mbodji	Dakar
Serbia	Vesna	Vekovic	Children's Hospital for Lung Diseases and Tb, University Hospital Dr Dragisa Misovic,Belgrade
Serbia	Zorica	Zivkovic	Children's Hospital for Lung Diseases and Tb, University Hospital Dr Dragisa Misovic, Belgrade; Faculty of Pharmacy in Novi Sad, University Business Academy Novi Sad
Somalia	Osman Muhyadin	Abdulle	Forlanini Hospital,National Tuberculosis Program and Somaville University, Mogadishu
South Africa	Brian	Allwood	Division of Pulmonology, Department of Medicine, Stellenbosch University; Tygerberg Hospital, Cape Town

South Sudan	Babiker	Adam	M-Pharma co Ltd
Sri Lanka	Sisira	Siribaddana	Rajarata University of Sri Lanka, Teaching Hospital Anuradhapura
Sudan	Rana	Ahmed	The Epidemiological Laboratory, Khartoum
Sudan	Mahdia	Elhadi	University of Hail
Sudan	Mohamed	Elmustafa	University of Gezira; Wad Medani College of Medical Sciences and Technology
Syrian Arab Republic	Yousser	Mohammad	Tishreen University, Latakia; Al Sham Private University, Damascus
Tanzania	Stellah	Mpagama	Kibong'oto Infectious Diseases Hospital
Tanzania	Bibie	Said	Kibong'oto Infectious Diseases Hospital
Thailand	Mongkol	Lao-Araya	Chiang Mai University Hospital, Faculty of Medicine, Chiang Mai University
Timor-Leste	Benilda Trias	de Gula	Saint Paul Clinic of the Sisters of St. Paul of Chartres Congregation
Tunisia	Agnes	Hamzaoui	Hopital A Mami, Ariana and Medicine School, Tunis
Turkiye	Kübra	Tunçel	University of Gazi
Turkiye	Islam	Sangac	
Turkiye	Tuğçe	Tayyar	Lavanta Pharmacy
Turkiye	Aygün	Gürgöze	Polatlı Can Hospital
Uganda	Rebecca	Nantanda	Makerere University Ung Institute, College of Health Sciences, Makerere University
United Kingdom	Jamilah	Meghji	National Heart & Lung Institute, Imperial College London
Venezuela	Maria	Montes de Oca	Universidad Central de Venezuela, Centro Medico de Caracas
Venezuela	Juan	Catari	Centro Medico de Caracas
Vietnam	Tran Thien Quan	Vu	University of Medicine and Pharmacy at Ho Chi Minh city
Yemen, Rep.	Mohammed	Mohammed	Al Taaon pharmacy
Yemen, Rep.	Ruba	Khaled	
Yemen, Rep.	Weiam	Hussein	

Zambia	Charles	Mataya Mphuka	Livingstone Central Hospital
Zimbabwe	Terrence	Rudado Musekiwa	Chimhanda District Hospital

Supplementary Table 3: STROBE Statement[1]

	Item No.	Recommendation	Page No.
**Title and abstract**	1	(*a*) Indicate the study's design with a commonly used term in the title or the abstract	1
		(*b*) Provide in the abstract an informative and balanced summary of what was done and what was found	1
**Introduction**			
Background/rationale	2	Explain the scientific background and rationale for the investigation being reported	2
Objectives	3	State specific objectives, including any prespecified hypotheses	2
**Methods**			
Study design	4	Present key elements of study design early in the paper	2, Appendix
Setting	5	Describe the setting, locations, and relevant dates, including periods of recruitment, exposure, follow-up, and data collection	2, Appendix
Participants	6	(*a*) *Cohort study*—Give the eligibility criteria, and the sources and methods of selection of participants. Describe methods of follow-up<br>*Case-control study*—Give the eligibility criteria, and the sources and methods of case ascertainment and control selection. Give the rationale for the choice of cases and controls<br>*Cross-sectional study*—Give the eligibility criteria, and the sources and methods of selection of participants	2, Appendix
		(*b*) *Cohort study*—For matched studies, give matching criteria and number of exposed and unexposed<br>*Case-control study*—For matched studies, give matching criteria and the number of controls per case	N/A

	Item No.	Recommendation	Page No.
Variables	7	Clearly define all outcomes, exposures, predictors, potential confounders, and effect modifiers. Give diagnostic criteria, if applicable	2, Appendix
Data sources/ measurement	8*	For each variable of interest, give sources of data and details of methods of assessment (measurement). Describe comparability of assessment methods if there is more than one group	Appendix
Bias	9	Describe any efforts to address potential sources of bias	Appendix
Study size	10	Explain how the study size was arrived at	Appendix
Quantitative variables	11	Explain how quantitative variables were handled in the analyses. If applicable, describe which groupings were chosen and why	2, Appendix
Statistical methods	12	(*a*) Describe all statistical methods, including those used to control for confounding	2, appendix
		(*b*) Describe any methods used to examine subgroups and interactions	N/A
		(*c*) Explain how missing data were addressed	Appendix
		(*d*) *Cohort study*—If applicable, explain how loss to follow-up was addressed<br>*Case-control study*—If applicable, explain how matching of cases and controls was addressed<br>*Cross-sectional study*—If applicable, describe analytical methods taking account of sampling strategy	N/A
		(*e*) Describe any sensitivity analyses	N/A
Participants	13*	(a) Report numbers of individuals at each stage of study—eg numbers potentially eligible, examined for eligibility, confirmed eligible, included in the study, completing follow-up, and analysed	2, appendix
		(b) Give reasons for non-participation at each stage	N/A

	Item No.	Recommendation	Page No.
		(c) Consider use of a flow diagram	N/A
Descriptive data	14*	(a) Give characteristics of study participants (eg demographic, clinical, social) and information on exposures and potential confounders	2, Appendix
		(b) Indicate number of participants with missing data for each variable of interest	Appendix
		(c) *Cohort study*—Summarise follow-up time (eg, average and total amount)	N/A
Outcome data	15*	*Cohort study*—Report numbers of outcome events or summary measures over time	
		Case-control study—Report numbers in each exposure category, or summary measures of exposure	
		Cross-sectional study—Report numbers of outcome events or summary measures	3-5, Appendix
Main results	16	(*a*) Give unadjusted estimates and, if applicable, confounder-adjusted estimates and their precision (eg, 95% confidence interval). Make clear which confounders were adjusted for and why they were included	3-5, Appendix
		(*b*) Report category boundaries when continuous variables were categorized	3-5, Appendix
		(*c*) If relevant, consider translating estimates of relative risk into absolute risk for a meaningful time period	N/A
Other analyses	17	Report other analyses done—eg analyses of subgroups and interactions, and sensitivity analyses	3-5, Appendix
Key results	18	Summarise key results with reference to study objectives	6-7

	Item No.	Recommendation	Page No.
Limitations	19	Discuss limitations of the study, taking into account sources of potential bias or imprecision. Discuss both direction and magnitude of any potential bias	6-7
Interpretation	20	Give a cautious overall interpretation of results considering objectives, limitations, multiplicity of analyses, results from similar studies, and other relevant evidence	6-7
Generalisability	21	Discuss the generalisability (external validity) of the study results	6-7
**Other information**			
Funding	22	Give the source of funding and the role of the funders for the present study and, if applicable, for the original study on which the present article is based	7

Information on the STROBE Initiative is available at www.strobe-statement.org.

Supplementary Table 4: Overview of included LMICs and facilities by income group and WHO region

LMIC: Low-income and middle-income country; WHO: World Health Organization; HCF: Healthcare facility; CMS: Central medicine stores; No information: NIA available; NGO: Non-governmental organisation.

(1) Data for 3 private pharmacies submitted; (2) Data for 2 private pharmacies submitted; (3) Data for 2 private pharmacies submitted; (4) Data for 3 HCFs submitted, 2 public and 1 private HCF; (5) Data for 2 public HCFs submitted; (6) Data for 3 pharmacies submitted, 2 pubic and 1 private pharmacy.

**Country**	**World Bank Income Group**[6]	**WHO Region**[9]	**Pharmacy – type of facility**	**HCF – type of facility**	**CMS – type of facility**
Albania	Upper middle income	Europe	Private	Public	NIA
Algeria	Lower middle income	Africa	Private	Public	CMS
Angola	Lower middle income	Africa	Private	Public	CMS
Argentina	Upper middle income	Americas	Private	Public	CMS
Bosnia and Herzegovina	Upper middle income	Europe	Private	Public	NIA
Brazil	Upper middle income	Americas	Private	Public	CMS
Burkina Faso	Low income	Africa	Private	Public	NIA
Cameroon	Lower middle income	Africa	Private	Public	CMS
Chad	Low income	Africa	NIA	NIA	CMS
China	Upper middle income	Western Pacific	Private (1)	Public	CMS
Democratic Republic of Congo	Low income	Africa	Private	Public	CMS
Ecuador	Upper middle income	Americas	Private	Private	CMS
Egypt	Lower middle income	Eastern Mediterranean	Private (2)	Private	CMS
Eswatini	Lower middle income	Africa	Private	NIA	CMS
Ethiopia	Low income	Africa	Private	Public	CMS
Ghana	Lower middle income	Africa	Private	Public	CMS
Guinea	Low income	Africa	Private	NIA	CMS
Honduras	Lower middle income	Americas	Private	Public	NIA
India	Lower middle income	South-East Asia	Private	Public	CMS

Indonesia	Lower middle income	South-East Asia	Private (3)	Private & Public (4)	CMS
Iran	Lower middle income	Eastern Mediterranean	Private	Public	CMS
Iraq	Upper middle income	Eastern Mediterranean	Private	Public	NIA
Kenya	Lower middle income	Africa	Private	Public	NIA
Kyrgyzstan	Lower middle income	Europe	Private	Public	CMS
Lesotho	Lower middle income	Africa	NIA	NGO	CMS
Libya	Upper middle income	Eastern Mediterranean	Private	Public	CMS
Malawi	Low income	Africa	Private	Public (5)	CMS
Malaysia	Upper middle income	Western Pacific	Private	Public	CMS
Mali	Low income	Africa	Private	Public	CMS
Mexico	Upper middle income	Americas	Private	Private	NIA
Moçambique	Low income	Africa	Private	Public	CMS
Mongolia	Lower middle income	Western Pacific	Private & Public (6)	Public	CMS
Nepal	Lower middle income	South-East Asia	Private	Public	CMS
Niger	Low income	Africa	Private	Public	CMS
Nigeria	Lower middle income	Africa	Private	Public	CMS
North Macedonia	Upper middle income	Europe	Public	Public	CMS
Pakistan	Lower middle income	Eastern Mediterranean	Public	Public	CMS
Peru	Upper middle income	Americas	Private	Public	CMS
Romania	Upper middle income	Europe	Private	Public	CMS
Rwanda	Low income	Africa	Private	Public	NIA
Senegal	Lower middle income	Africa	Private	Public	CMS
Serbia	Upper middle income	Europe	Private	Public	CMS
Somalia	Low income	Eastern Mediterranean	Private	Private	NIA
South Africa	Upper middle income	Africa	Private	Public	CMS
South Sudan	Low income	Africa	Private	NIA	NIA
Sri Lanka	Lower middle income	South-East Asia	Private	Public	CMS
Sudan	Low income	Eastern Mediterranean	Private	Public	CMS

Syria	Low income	Eastern Mediterranean	Private	Public	CMS
Tanzania	Lower middle income	Africa	Private	Public	CMS
Thailand	Upper middle income	South-East Asia	Public	Public	NIA
The Gambia	Low income	Africa	Private	Public	CMS
Timor Leste	Lower middle income	South-East Asia	NIA	Private	NIA
Tunisia	Lower middle income	Eastern Mediterranean	Public	Public	CMS
Turkiye	Upper middle income	Europe	Private	Private	CMS
Uganda	Low income	Africa	Private	NGO	CMS
Venezuela		Americas	Private	Private	NIA
Viet Nam	Lower middle income	Western Pacific	Private	Public	CMS
Yemen	Low income	Eastern Mediterranean	Public	Private	NIA
Zambia	Low income	Africa	Private	Public	CMS
Zimbabwe	Lower middle income	Africa	Private	Public	CMS

Supplementary table 5: Availability, Cost and affordability of Nicotine gums in pharmacies, HCFs and CMSs in LMIC

Cost is for standardised dose (10 gums/day for 30 days). Affordable: one month's treatment costs <1 day's wage of national minimum wage. $: US$; HCF: Healthcare facility; CMS: Central medicine stores; NIA: no information available; DRC: Democratic Republic of Congo; NGO: Non-governmental organisation; DOW: Days of work

		PHARMACY						HCF						CMS		
**Country**	**Minimum daily wage (US$)**	**Type of facility**	**Gum available?**	**Strength (pack size)**	**Price 10 gums/day for 30 days ($)**	**DOW to pay 1 month wage**	**Gum affordable?**	**Type of facility**	**Gum available?**	**Strength (pack size)**	**Price 10 gums/day for 30 days ($)**	**DOW for 1 month supply**	**Gum affordable?**	**Gum available?**	**Strength (pack size)**	**Price 10 gums/day for 30 days ($)**
Albania	11.09	Private	Unavailable	Unavailable	Unavailable		Unavailable	Public	Unavailable	Unavailable	Unavailable		Unavailable	NIA	NIA	NIA
Algeria	5.39	Private	Unavailable	Unavailable	Unavailable		Unavailable	Public	Unavailable	Unavailable	Unavailable		Unavailable	Unavailable	Unavailable	Unavailable
Angola	2.46	Private	Unavailable	Unavailable	Unavailable		Unavailable	Public	Unavailable	Unavailable	Unavailable		Unavailable	Unavailable	Unavailable	Unavailable
Argentina	15.60	Private	Available	2 mg, (24 gums)	161.53	10.35	Unaffordable	Public	Unavailable	Unavailable	Unavailable		Unavailable	Unavailable	Unavailable	Unavailable
Bosnia and Herzegovin a	10.89	Private	Available	2 mg, (30 gums)	65.13	5.98	Unaffordable	Public	Available	2 mg (20 gums)	97.69	8.97	Unaffordable	NIA	NIA	NIA
Brazil	8.86	Private	Available	2 mg (30 gums)	120.44	13.59	Unaffordable	Public	Unavailable	Unavailable	Unavailable		Unavailable	Unavailable	NIA	Unavailable
Burkina Faso	2.13	Private	Available	2 mg (30 gums)	136.00	63.71	Unaffordable	Public	Unavailable	Unavailable	Unavailable		Unavailable	NIA	NIA	NIA
Cameroon	2.23	Private	Available	4 mg (60 gums)	96.00	42.98	Unaffordable	Public	Unavailable	Unavailable	Unavailable		Unavailable	Unavailable	Unavailable	Unavailable
Chad	3.70	NIA	NIA	NIA	NIA		NIA	NIA	NIA	NIA	NIA		NIA	Unavailable	Unavailable	Unavailable
China	10.62	Private	Available	2 mg, (30 gums)	104.39	9.83	Unaffordable	Public	Unavailable	Unavailable	Unavailable		Unavailable	Unavailable	Unavailable	Unavailable

Democratic Republic of Congo	3.54	Private	Unavailable	Unavailable	Unavailable		Unavailable	Public	Unavailable	Unavailable	Unavailable		Unavailable	Unavailable	Unavailable	Unavailable
Ecuador	15.17	Private	Unavailable	Unavailable	Unavailable		Unavailable	Private	Unavailable	Unavailable	Unavailable		Unavailable	Unavailable	Unavailable	Unavailable
Egypt	5.85	Private	Available	4 mg (10 gums)	28.90	4.94	Unaffordable	Private	Unavailable	Unavailable	Unavailable		Unavailable	Unavailable	Unavailable	Unavailable
Eswatini	0.92	Private	Unavailable	Unavailable	Unavailable		Unavailable	NIA	NIA	NIA	NIA		NIA	Unavailable	Unavailable	Unavailable
Ethiopia	0.31	Private	Unavailable	Unavailable	Unavailable		Unavailable	Public	Unavailable	Unavailable	Unavailable		Unavailable	Unavailable	Unavailable	Unavailable
Ghana	1.19	Private	Unavailable	Unavailable	Unavailable		Unavailable	Public	Unavailable	Unavailable	Unavailable		Unavailable	Unavailable	Unavailable	Unavailable
Guinea	2.44	Private	Unavailable	Unavailable	Unavailable		Unavailable	NIA	NIA	NIA	NIA		NIA	Unavailable	Unavailable	Unavailable
Honduras	14.82	Private	Unavailable	Unavailable	Unavailable		Unavailable	Public	Unavailable	Unavailable	Unavailable		Unavailable	NIA	NIA	NIA
India	2.14	Private	Available	4 mg (9 gums/pack)	46.00	21.52	Unaffordable	Public	Unavailable	Unavailable	Unavailable		Unavailable	Unavailable	Unavailable	Unavailable
Indonesia	7.22	Private	Unavailable	No information	Unavailable		Unavailable	Public & Private	Unavailable	Unavailable	Unavailable		Unavailable	Unavailable	Unavailable	Unavailable
Iran	20.44	Private	Available	2 mg, (30 gums)	120.00	5.87	Unaffordable	Public	Unavailable	Unavailable	Unavailable		Unavailable	Available	2 mg (30 gums)	120.00
Iraq	9.43	Private	Unavailable	Unavailable	Unavailable		Unavailable	Public	Unavailable	Unavailable	Unavailable		Unavailable	NIA	NIA	NIA
Kenya	5.25	Private	Unavailable	Unavailable	Unavailable		Unavailable	Public	Unavailable	Unavailable	Unavailable		Unavailable	NIA	NIA	NIA
Kyrgyzstan	0.88	Private	Unavailable	Unavailable	Unavailable		Unavailable	Public	Unavailable	Unavailable	Unavailable		Unavailable	Unavailable	Unavailable	Unavailable
Lesotho	3.60	NIA	NIA	NIA	NIA		NIA	NGO	Available	2 mg (NIA)	NIA		NIA	Unavailable	Unavailable	Unavailable
Libya	3.59	Private	Available	4 mg (105 gums)	79.84	22.27	Unaffordable	Public	Unavailable	Unavailable	Unavailable		Unavailable	Unavailable	Unavailable	Unavailable
Malawi	1.92	Private	Unavailable	Unavailable	Unavailable		Unavailable	Public	Unavailable	Unavailable	Unavailable		Unavailable	Unavailable	Unavailable	Unavailable

Malaysia	12.88	Private	Available	2 mg (105 gums)	68.79	5.34	Unaffordable	Public	Unavailable	Unavailable	Unavailable		Unavailable	Unavailable	Unavailable	Unavailable
Mali	2.46	Private	Available	2 mg (30 gums)	166.16	67.45	Unaffordable	Public	Unavailable	Unavailable	Unavailable		Unavailable	Unavailable	Unavailable	Unavailable
Mexico	15.07	Private	Unavailable	Unavailable	Unavailable		Unavailable	Private	Unavailable	Unavailable	Unavailable		Unavailable	NIA	NIA	NIA
Moçambique	4.80	Private	Unavailable	Unavailable	Unavailable		Unavailable	Public	Unavailable	Unavailable	Unavailable		Unavailable	Unavailable	Unavailable	Unavailable
Mongolia	4.85	Public & Private	Unavailable	Unavailable	Unavailable		Unavailable	Public	Unavailable	Unavailable	Unavailable		Unavailable	Unavailable	Unavailable	Unavailable
Nepal	4.56	Private	Unavailable	Unavailable	Unavailable		Unavailable	Public	Unavailable	Unavailable	Unavailable		Unavailable	Unavailable	Unavailable	Unavailable
Niger	2.31	Private	Available	Other (36 gums)	123.98	53.60	Unaffordable	Public	Unavailable	Unavailable	Unavailable		Unavailable	Unavailable	Unavailable	Unavailable
Nigeria	2.77	Private	Available	2 mg (15 gums)	139.20	50.23	Unaffordable	Public	Unavailable	Unavailable	Unavailable		Unavailable	Unavailable	Unavailable	Unavailable
North Macedonia	17.37	Public	Unavailable	Unavailable	Unavailable		Unavailable	Public	Unavailable	Unavailable	Unavailable		Unavailable	Unavailable	Unavailable	Unavailable
Pakistan	4.23	Public	Unavailable	Unavailable	Unavailable		Unavailable	Public	Unavailable	Unavailable	Unavailable		Unavailable	Unavailable	Unavailable	Unavailable
Peru	9.90	Private	Unavailable	Unavailable	Unavailable		Unavailable	Public	Unavailable	Unavailable	Unavailable		Unavailable	Unavailable	Unavailable	Unavailable
Romania	20.32	Private	Available	2 mg (30 gums)	88.99	4.38	Unaffordable	Public	Available	2 mg (30 gums)	63.14	3.11	Unaffordable	Available	2mg (30 gums)	NIA
Rwanda	0.09	Private	Unavailable	Unavailable	Unavailable		Unavailable	Public	Unavailable	Unavailable	Unavailable		Unavailable	NIA	NIA	NIA
Senegal	3.34	Private	Unavailable	Unavailable	Unavailable		Unavailable	Public	Unavailable	Unavailable	Unavailable		Unavailable	Unavailable	Unavailable	Unavailable
Serbia	16.35	Private	Available	4 mg (30 gums)	NIA		NIA	Public	Available	4 mg (20 gums)	60.75	3.72	Unaffordable	Available	4 mg (30 gums)	NIA
Somalia	5.05	Private	Unavailable	Unavailable	Unavailable		Unavailable	Private	Unavailable	Unavailable	Unavailable		Unavailable	NIA	NIA	NIA
South Africa	9.37	Private	Available	4 mg (30 gums)	81.95	8.74	Unaffordable	Public	Unavailable	Unavailable	Unavailable		Unavailable	Unavailable	Unavailable	Unavailable
South Sudan	5.05	Private	Unavailable	Unavailable	Unavailable		Unavailable	NIA	NIA	NIA	NIA		NIA	NIA	NIA	NIA

Sri Lanka	1.35	Private	Unavailable	Unavailable	Unavailable		Unavailable	Public	Unavailable	Unavailable	Unavailable		Unavailable	Unavailable	Unavailable	Unavailable
Sudan	0.03	Private	Unavailable	Unavailable	Unavailable		Unavailable	Public	Unavailable	Unavailable	Unavailable		Unavailable	Unavailable	Unavailable	Unavailable
Syria	0.15	Private	Unavailable	Unavailable	Unavailable		Unavailable	Public	Unavailable	Unavailable	Unavailable		Unavailable	Unavailable	Unavailable	Unavailable
Tanzania	2.18	Private	Unavailable	Unavailable	Unavailable		Unavailable	Public	Unavailable	Unavailable	Unavailable		Unavailable	Unavailable	Unavailable	Unavailable
Thailand	9.05	Public	Unavailable	Unavailable	Unavailable		Unavailable	Public	Unavailable	Unavailable	Unavailable		Unavailable	NIA	NIA	NIA
The Gambia	0.91	Private	Available	4 mg (15 gums)	109.20	119.91	Unaffordable	Public	Unavailable	Unavailable	Unavailable		Unavailable	Unavailable	Unavailable	Unavailable
Timor Leste	4.43	NIA	NIA	NIA	NIA		NIA	Private	Unavailable	Unavailable	Unavailable		Unavailable	NIA	NIA	NIA
Tunisia	4.74	Public	Unavailable	Unavailable	Unavailable		Unavailable	Public	Unavailable	Unavailable	Unavailable		Unavailable	Unavailable	Unavailable	Unavailable
Turkiye	13.90	Private	Available	2 mg (30 gums)	52.11	3.75	Unaffordable	Private	Available	4 mg (30 gums)	54.26	3.90	Unaffordable	Available	2 mg (105 gums)	11.44
Uganda	0.07	Private	Unavailable	Unavailable	Unavailable		Unavailable	NGO	Unavailable	Unavailable	Unavailable		Unavailable	Unavailable	Unavailable	Unavailable
Venezuela	0.94	Private	Unavailable	Unavailable	Unavailable		Unavailable	Private	Unavailable	Unavailable	Unavailable		Unavailable	NIA	NIA	NIA
Viet Nam	6.00	Private	Unavailable	Unavailable	Unavailable		Unavailable	Public	Unavailable	Unavailable	Unavailable		Unavailable	Unavailable	Unavailable	Unavailable
Yemen	3.23	Public	Unavailable	Unavailable	Unavailable		Unavailable	Private	Unavailable	Unavailable	Unavailable		Unavailable	NIA	NIA	NIA
Zambia	2.59	Private	Available	2 mg (105 gums)	20.11	7.78	Unaffordable	Public	Unavailable	Unavailable	Unavailable		Unavailable	Unavailable	Unavailable	Unavailable
Zimbabwe	11.58	Private	Unavailable	Unavailable	Unavailable		Unavailable	Public	Unavailable	Unavailable	Unavailable		Unavailable	Unavailable	Unavailable	Unavailable

Supplementary Table 6: Availability, cost and affordability of nicotine transdermal patches in pharmacies, HCFs and CMSs in LMIC

Cost is for standardised dose (10 gums/day for 30 days). Affordable: one month's treatment costs <1 day's wage of national minimum wage. $: US$; HCF: Healthcare facility; CMS: Central medicine stores; NIA: no information available; DRC: Democratic Republic of Congo; NGO: Non-governmental organisation; DOW: Days Of Work

		Pharmacy						HCF						CMS		
Country	Minimum daily wage (US$)	Type of facility	Patches Available?	Strength (pack size)	Price 30 patches ($)	Dow for 1 month treatment	Affordable?	Type of facility	Patches Available?	Strength	Price 30 patches ($)	DOW for 1 mth supply	Affordable?	Available?	Strength (pack size)	Price 30 patches ($)
Albania	11.09	Private	Unavailable	Unavailable	Unavailable		Unavailable	Public	Unavailable	Unavailable	Unavailable		Unavailable	NIA	NIA	NIA
Algeria	5.39	Private	Unavailable	Unavailable	Unavailable		Unavailable	Public	Unavailable	Unavailable	Unavailable		Unavailable	Unavailable	Unavailable	Unavailable
Angola	2.46	Private	Unavailable	Unavailable	Unavailable		Unavailable	Public	Unavailable	Unavailable	Unavailable		Unavailable	Unavailable	Unavailable	Unavailable
Argentina	15.60	Private	Unavailable	Unavailable	Unavailable		Unavailable	Public	Unavailable	Unavailable	Unavailable		Unavailable	Unavailable	Unavailable	Unavailable
Bosnia and Herzegovina	10.89	Private	Available	15 mg (7 patches)	63.64	5.84	Unaffordable	Public	Available	15 mg / 25 mg (1 patch)	468.90	43.06	Unaffordable	NIA	NIA	NIA
Brazil	8.86	Private	Available	14 mg (7 patches)	69.21	7.81	Unaffordable	Public	Unavailable	Unavailable	Unavailable		Unavailable	Unavailable	Unavailable	Unavailable
Burkina Faso	2.13	Private	Unavailable	Unavailable	Unavailable		Unavailable	Public	Unavailable	Unavailable	Unavailable		Unavailable	NIA	NIA	NIA
Cameroon	2.23	Private	Unavailable	Unavailable	Unavailable		Unavailable	Public	Unavailable	Unavailable	Unavailable		Unavailable	Unavailable	Unavailable	Unavailable
Chad	3.70	NIA	NIA	NIA	Unavailable		Unavailable	NIA	NIA	NIA	NIA		NIA	Unavailable	Unavailable	Unavailable
China	10.62	Private	Available	NIA (10 patches)	4.93	0.46	Affordable	Public	Unavailable	Unavailable	Unavailable		Unavailable	Unavailable	Unavailable	Unavailable
Democratic Republic of Congo	3.54	Private	Unavailable	Unavailable	Unavailable		Unavailable	Public	Unavailable	Unavailable	Unavailable		Unavailable	Unavailable	Unavailable	Unavailable
Ecuador	15.17	Private	Unavailable	Unavailable	Unavailable		Unavailable	Private	Unavailable	Unavailable	Unavailable		Unavailable	Unavailable	Unavailable	Unavailable

Egypt	5.85	Private	Available	7 mg / 14 mg / 21mg (1 patch)	12.17	2.08	Unaffordable	Private	Unavailable	Unavailable	Unavailable		Unavailable	Unavailable	Unavailable	Unavailable
Eswatini	0.92	Private	Unavailable	Unavailable	Unavailable		Unavailable	NIA	NIA	NIA	NIA		NIA	Unavailable	Unavailable	Unavailable
Ethiopia	0.31	Private	Unavailable	Unavailable	Unavailable		Unavailable	Public	Unavailable	Unavailable	Unavailable		Unavailable	Unavailable	Unavailable	Unavailable
Ghana	1.19	Private	Unavailable	Unavailable	Unavailable		Unavailable	Public	Unavailable	Unavailable	Unavailable		Unavailable	Unavailable	Unavailable	Unavailable
Guinea	2.44	Private	Unavailable	Unavailable	Unavailable		Unavailable	NIA	NIA	NIA	NIA		NIA	Unavailable	Unavailable	Unavailable
Honduras	14.82	Private	Unavailable	Unavailable	Unavailable		Unavailable	Public	Unavailable	Unavailable	Unavailable		Unavailable	NIA	NIA	NIA
India	2.14	Private	Unavailable	Unavailable	Unavailable		Unavailable	Public	Unavailable	Unavailable	Unavailable		Unavailable	Unavailable	Unavailable	Unavailable
Indonesia	7.22	Private	Unavailable	Unavailable	Unavailable		Unavailable	Public & Private	Unavailable	Unavailable	Unavailable		Unavailable	Unavailable	Unavailable	Unavailable
Iran	20.44	Private	Unavailable	Unavailable	Unavailable		Unavailable	Public	Unavailable	Unavailable	Unavailable		Unavailable	Unavailable	Unavailable	Unavailable
Iraq	9.43	Private	Unavailable	Unavailable	Unavailable		Unavailable	Public	Unavailable	Unavailable	Unavailable		Unavailable	NIA	NIA	NIA
Kenya	5.25	Private	Unavailable	Unavailable	Unavailable		Unavailable	Public	Available	1 mg (7 patches)	145.71	27.78	Unaffordable	NIA	NIA	NIA
Kyrgyzstan	0.88	Private	Unavailable	Unavailable	Unavailable		Unavailable	Public	Available	NIA (7 patches)	68.61	77.99	Unaffordable	Available	NIA (7 patches)	124.29
Lesotho	3.60	No information	NIA	NIA	NIA		NIA	NGO	Available	NIA	NIA		NIA	Unavailable	Unavailable	Unavailable
Libya	3.59	Private	Unavailable	Unavailable	Unavailable		Unavailable	Public	Unavailable	Unavailable	Unavailable		Unavailable	Unavailable	Unavailable	Unavailable
Malawi	1.92	Private	Unavailable	Unavailable	Unavailable		Unavailable	Public	Unavailable	Unavailable	Unavailable		Unavailable	Unavailable	Unavailable	Unavailable
Malaysia	12.88	Private	Available	10 mg (7 patches)	57.52	4.47	Unaffordable	Public	Unavailable	Unavailable	Unavailable		Unavailable	Unavailable	Unavailable	Unavailable
Mali	2.46	Private	Unavailable	Unavailable	Unavailable		Unavailable	Public	Unavailable	Unavailable	Unavailable		Unavailable	Unavailable	Unavailable	Unavailable
Mexico	15.07	Private	Available	21 mg (7)	49.07	3.26	Unaffordable	Private	Unavailable	Unavailable	Unavailable		Unavailable	NIA	NIA (NIA)	NIA
Moçambique	4.80	Private	Unavailable	Unavailable	Unavailable		Unavailable	Public	Unavailable	Unavailable	Unavailable		Unavailable	Unavailable	Unavailable	Unavailable

Mongolia	4.85	Public & Private	Available	NIA (28)	54.00	11.13	Unaffordable	Public	Unavailable	Unavailable	Unavailable		Unavailable	Available	NIA (30)	12.00
Nepal	4.56	Private	Unavailable	Unavailable	Unavailable		Unavailable	Public	Unavailable	Unavailable	Unavailable		Unavailable	Unavailable	Unavailable	Unavailable
Niger	2.31	Private	Available	15 mg (1)	437.94	189.33	Unaffordable	Public	Unavailable	Unavailable	Unavailable		Unavailable	Unavailable	Unavailable	Unavailable
Nigeria	2.77	Private	Unavailable	Unavailable	Unavailable		Unavailable	Public	Unavailable	Unavailable	Unavailable		Unavailable	Unavailable	Unavailable	Unavailable
North Macedonia	17.37	Public	Unavailable	Unavailable	Unavailable		Unavailable	Public	Unavailable	Unavailable	Unavailable		Unavailable	Unavailable	Unavailable	Unavailable
Pakistan	4.23	Public	Unavailable	Unavailable	Unavailable		Unavailable	Public	Unavailable	Unavailable	Unavailable		Unavailable	Unavailable	Unavailable	Unavailable
Peru	9.90	Private	Unavailable	Unavailable	Unavailable		Unavailable	Public	Unavailable	Unavailable	Unavailable		Unavailable	Unavailable	Unavailable	Unavailable
Romania	20.32	Private	Available	15 mg (7)	62.09	3.06	Unaffordable	Public	Unavailable	Unavailable	Unavailable		Unavailable	Available	10 mg (7)	NIA
Rwanda	0.09	Private	Unavailable	Unavailable	Unavailable		Unavailable	Public	Unavailable	Unavailable	Unavailable		Unavailable	NIA	NIA	NIA
Senegal	3.34	Private	Unavailable	Unavailable	Unavailable		Unavailable	Public	Unavailable	Unavailable	Unavailable		Unavailable	Unavailable	Unavailable	Unavailable
Serbia	16.35	Private	Unavailable	Unavailable	Unavailable		Unavailable	Public	Available	25 mg (7)	25.57	1.57	Unaffordable	Available	10 mg (7)	NIA
Somalia	5.05	Private	Unavailable	Unavailable	Unavailable		Unavailable	Private	Unavailable	Unavailable	Unavailable		Unavailable	NIA	NIA	NIA
South Africa	9.37	Private	Available	10 mg (7)	68.57	7.32	Unaffordable	Public	Unavailable	Unavailable	Unavailable		Unavailable	Unavailable	Unavailable	Unavailable
South Sudan	5.05	Private	Unavailable	Unavailable	Unavailable		Unavailable	No information	NIA	NIA	NIA		NIA	NIA	NIA	NIA
Sri Lanka	1.35	Private	Unavailable	Unavailable	Unavailable		Unavailable	Public	Unavailable	Unavailable	Unavailable		Unavailable	Unavailable	Unavailable	Unavailable
Sudan	0.03	Private	Unavailable	Unavailable	Unavailable		Unavailable	Public	Unavailable	Unavailable	Unavailable		Unavailable	Unavailable	Unavailable	Unavailable
Syria	0.15	Private	Unavailable	Unavailable	Unavailable		Unavailable	Public	Unavailable	Unavailable	Unavailable		Unavailable	Unavailable	Unavailable	Unavailable
Tanzania	2.18	Private	Unavailable	Unavailable	Unavailable		Unavailable	Public	Unavailable	Unavailable	Unavailable		Unavailable	Unavailable	Unavailable	Unavailable
Thailand	9.05	Public	Unavailable	Unavailable	Unavailable		Unavailable	Public	Unavailable	Unavailable	Unavailable		Unavailable	NIA	NIA	NIA
The Gambia	0.91	Private	Available	15 mg (7 pathces)	132.60	145.60	Unaffordable	Public	Unavailable	Unavailable	Unavailable		Unavailable	Unavailable	Unavailable	Unavailable
Timor Leste	4.43	No information	NIA	NIA	NIA		NIA	Private	Unavailable	Unavailable	Unavailable		Unavailable	NIA	NIA	NIA

Tunisia	4.74	Public	Unavailable	Unavailable	Unavailable		Unavailable	Public	Unavailable	Unavailable	Unavailable		Unavailable	Unavailable	Unavailable	Unavailable
Turkiye	13.90	Private	Available	15 mg (7 patches)	62.03	4.46	Unaffordable	Private	Available	25 mg (7 patches)	62.03	4.46	Unaffordable	Available	15 mg (7 patches)	43.72
Uganda	0.07	Private	Unavailable	Unavailable	Unavailable		Unavailable	NGO	Unavailable	Unavailable	Unavailable		Unavailable	Unavailable	Unavailable	Unavailable
Venezuela	0.94	Private	Unavailable	Unavailable	Unavailable		Unavailable	Private	Unavailable	Unavailable	Unavailable		Unavailable	NIA	NIA	NIA
Viet Nam	6.00	Private	Unavailable	Unavailable	Unavailable		Unavailable	Public	Unavailable	Unavailable	Unavailable		Unavailable	Unavailable	Unavailable	Unavailable
Yemen	3.23	Public	Unavailable	Unavailable	Unavailable		Unavailable	Private	Unavailable	Unavailable	Unavailable		Unavailable	NIA	NIA	NIA
Zambia	2.59	Private	Available	15 mg (7 patches)	178.29	68.93	Unaffordable	Public	Unavailable	Unavailable	Unavailable		Unavailable	Unavailable	Unavailable	Unavailable
Zimbabwe	11.58	Private	Unavailable	Unavailable	Unavailable		Unavailable	Public	Unavailable	Unavailable	Unavailable		Unavailable	Unavailable	Unavailable	Unavailable

Supplementary Table 7: Availability, cost and affordability of Bupropion tablets in pharmacies, HCFs and CMSs in LMIC

Cost is for standardised dose (10 gums/day for 30 days). Affordable: one month's treatment costs <1 day's wage of national minimum wage. $: US$; HCF: Healthcare facility; CMS: Central medicine stores; NIA: no information available; DRC: Democratic Republic of Congo; NGO: Non-governmental organisation.

		Pharmacy							HCF							CMS			
Country	Minimum daily wage (US$)	Type of facility	Tablet Available?	Drug	Strength (pack size)	Price 60 tablets ($)	DOW for 1 month supply	Affordable?	Type of facility	Available?	Drug	Strength (pack size)	Price of 60 tablets ($)	DOW for 1 month supply	Affordable?	Available?	Drug	Strength (pack size)	Price 60 tablets ($)
Albania	11.09	Private	Unavailable	Unavailable	Unavailable	Unavailable		Unavailable	Public	Unavailable	Unavailable	Unavailable	Unavailable		Unavailable	NIA	NIA	NIA	NIA
Algeria	5.39	Private	Unavailable	Unavailable	Unavailable	Unavailable		Unavailable	Public	Unavailable	Unavailable	Unavailable	Unavailable		Unavailable	Unavailable	Unavailable	Unavailable	Unavailable
Angola	2.46	Private	Unavailable	Unavailable	Unavailable	Unavailable		Unavailable	Public	Unavailable	Unavailable	Unavailable	Unavailable		Unavailable	Unavailable	Unavailable	Unavailable	Unavailable
Argentina	15.60	Private	Unavailable	Unavailable	Unavailable	Unavailable		Unavailable	Public	Unavailable	Unavailable	Unavailable	Unavailable		Unavailable	Unavailable	Unavailable	Unavailable	Unavailable
Bosnia and Herzegovina	10.89	Private	Unavailable	Unavailable	Unavailable	Unavailable		Unavailable	Public	Unavailable	Unavailable	Unavailable	Unavailable		Unavailable	NIA	NIA	NIA	NIA
Brazil	8.86	Private	Unavailable	Unavailable	Unavailable	Unavailable		Unavailable	Public	Unavailable	Unavailable	Unavailable	Unavailable		Unavailable	Unavailable	Unavailable	Unavailable	Unavailable
Burkina Faso	2.13	Private	Unavailable	Unavailable	Unavailable	Unavailable		Unavailable	Public	Unavailable	Unavailable	Unavailable	Unavailable		Unavailable	NIA	NIA	NIA	NIA
Cameroon	2.23	Private	Unavailable	Unavailable	Unavailable	Unavailable		Unavailable	Public	Unavailable	Unavailable	Unavailable	Unavailable		Unavailable	Unavailable	Unavailable	Unavailable	Unavailable
Chad	3.70	NIA	NIA	NIA	NIA	NIA		NIA	NIA	NIA	NIA	NIA	NIA		NIA	Unavailable	Unavailable	Unavailable	Unavailable
China	10.62	Private	Unavailable	Unavailable	Unavailable	Unavailable		Unavailable	Public	Unavailable	Unavailable	Unavailable	Unavailable		Unavailable	Unavailable	Unavailable	Unavailable	Unavailable
Democratic Republic of Congo	3.54	Private	Unavailable	Unavailable	Unavailable	Unavailable		Unavailable	Public	Unavailable	Unavailable	Unavailable	Unavailable		Unavailable	Unavailable	Unavailable	Unavailable	Unavailable

Ecuador	15.17	Private	Unavailable	Unavailable	Unavailable	Unavailable		Unavailable	Private	Unavailable	Unavailable	Unavailable	Unavailable		Unavailable	Unavailable	Unavailable	Unavailable	Unavailable
Egypt	5.85	Private	Unavailable	Unavailable	Unavailable	Unavailable		Unavailable	Private	Unavailable	Unavailable	Unavailable	Unavailable		Unavailable	Unavailable	Unavailable	Unavailable	Unavailable
Eswatini	0.92	Private	Unavailable	Unavailable	Unavailable	Unavailable		Unavailable	NIA	NIA	NIA	NIA	NIA		NIA	Unavailable	Unavailable	Unavailable	Unavailable
Ethiopia	0.31	Private	Unavailable	Unavailable	Unavailable	Unavailable		Unavailable	Public	Unavailable	Unavailable	Unavailable	Unavailable		Unavailable	Unavailable	Unavailable	Unavailable	Unavailable
Ghana	1.19	Private	Unavailable	Unavailable	Unavailable	Unavailable		Unavailable	Public	Unavailable	Unavailable	Unavailable	Unavailable		Unavailable	Unavailable	Unavailable	Unavailable	Unavailable
Guinea	2.44	Private	Unavailable	Unavailable	Unavailable	Unavailable		Unavailable	NIA	NIA	NIA	NIA	NIA		NIA	Unavailable	Unavailable	Unavailable	Unavailable
Honduras	14.82	Private	Unavailable	Unavailable	Unavailable	Unavailable		Unavailable	Public	Unavailable	Unavailable	Unavailable	Unavailable		Unavailable	NIA	NIA	NIA	NIA
India	2.14	Private	Unavailable	Unavailable	Unavailable	Unavailable		Unavailable	Public	Unavailable	Unavailable	Unavailable	Unavailable		Unavailable	Unavailable	Unavailable	Unavailable	Unavailable
Indonesia	7.22	Private	Unavailable	Unavailable	Unavailable	Unavailable		Unavailable	Public & Private	Unavailable	Unavailable	Unavailable	Unavailable		Unavailable	Unavailable	Unavailable	Unavailable	Unavailable
Iran	20.44	Private	Unavailable	Unavailable	Unavailable	Unavailable		Unavailable	Public	Unavailable	Unavailable	Unavailable	Unavailable		Unavailable	Unavailable	Unavailable	Unavailable	Unavailable
Iraq	9.43	Private	Unavailable	Unavailable	Unavailable	Unavailable		Unavailable	Public	Unavailable	Unavailable	Unavailable	Unavailable		Unavailable	NIA	NIA	NIA	NIA
Kenya	5.25	Private	Unavailable	Unavailable	Unavailable	Unavailable		Unavailable	Public	Unavailable	Unavailable	Unavailable	Unavailable		Unavailable	NIA	NIA	NIA	NIA
Kyrgyzstan	0.88	Private	Unavailable	Unavailable	Unavailable	Unavailable		Unavailable	Public	Unavailable	Unavailable	Unavailable	Unavailable		Unavailable	Unavailable	Unavailable	Unavailable	Unavailable
Lesotho	3.60	No information	NIA	NIA	NIA	NIA		NIA	NGO	Unavailable	Unavailable	Unavailable	Unavailable		Unavailable	Unavailable	Unavailable	Unavailable	Unavailable
Libya	3.59	Private	Unavailable	Unavailable	Unavailable	Unavailable		Unavailable	Public	Unavailable	Unavailable	Unavailable	Unavailable		Unavailable	Unavailable	Unavailable	Unavailable	Unavailable
Malawi	1.92	Private	Unavailable	Unavailable	Unavailable	Unavailable		Unavailable	Public	Unavailable	Unavailable	Unavailable	Unavailable		Unavailable	Unavailable	Unavailable	Unavailable	Unavailable
Malaysia	12.88	Private	Unavailable	Unavailable		Unavailable		Unavailable	Public	Unavailable	Unavailable	Unavailable	Unavailable		Unavailable	Unavailable	Unavailable	Unavailable	Unavailable
Mali	2.46	Private	Unavailable	Unavailable	Unavailable	Unavailable		Unavailable	Public	Unavailable	Unavailable	Unavailable	Unavailable		Unavailable	Unavailable	Unavailable	Unavailable	Unavailable
Mexico	15.07	Private	Unavailable	Unavailable	Unavailable	Unavailable		Unavailable	Private	Unavailable	Unavailable	Unavailable	Unavailable		Unavailable	NIA	NIA	NIA	NIA
Moçambique	4.80	Private	Unavailable	Unavailable	Unavailable	Unavailable		Unavailable	Public	Unavailable	Unavailable	Unavailable	Unavailable		Unavailable	Unavailable	Unavailable	Unavailable	Unavailable

Mongolia	4.85	Public & Private	Unavailable	Unavailable	Unavailable	Unavailable		Unavailable	Public	Unavailable	Unavailable	Unavailable	Unavailable		Unavailable	Unavailable	Unavailable	Unavailable	Unavailable
Nepal	4.56	Private	Unavailable	Unavailable	Unavailable	Unavailable		Unavailable	Public	Unavailable	Unavailable	Unavailable	Unavailable		Unavailable	Unavailable	Unavailable	Unavailable	Unavailable
Niger	2.31	Private	Unavailable	Unavailable	Unavailable	Unavailable		Unavailable	Public	Unavailable	Unavailable	Unavailable	Unavailable		Unavailable	Unavailable	Unavailable	Unavailable	Unavailable
Nigeria	2.77	Private	Available	Bupropion	150 mg (60 tablets)	158.40	57.16	Unaffordable	Public	Unavailable	Unavailable	Unavailable	Unavailable		Unavailable	Unavailable	Unavailable	Unavailable	Unavailable
North Macedonia	17.37	Public	Unavailable	Unavailable	Unavailable	Unavailable		Unavailable	Public	Unavailable	Unavailable	Unavailable	Unavailable		Unavailable	Unavailable	Unavailable	Unavailable	Unavailable
Pakistan	4.23	Public	Unavailable	Unavailable	Unavailable	Unavailable		Unavailable	Public	Unavailable	Unavailable	Unavailable	Unavailable		Unavailable	Unavailable	Unavailable	Unavailable	Unavailable
Peru	9.90	Private	Unavailable	Unavailable	Unavailable	Unavailable		Unavailable	Public	Unavailable	Unavailable	Unavailable	Unavailable		Unavailable	Unavailable	Unavailable	Unavailable	Unavailable
Romania	20.32	Private	Available	Bupropion	150 mg (30 tablets)	129.38	6.37	Unaffordable	Public	Available	Bupropion	150 mg (30 tablets)	26.78	1.32	Unaffordable	Available	Bupropion	150 mg (NIA)	NIA
Rwanda	0.09	Private	Unavailable	Unavailable	Unavailable	Unavailable		Unavailable	Public	Unavailable	Unavailable	Unavailable	Unavailable		Unavailable	NIA	NIA	NIA	NIA
Senegal	3.34	Private	Unavailable	Unavailable	Unavailable	Unavailable		Unavailable	Public	Unavailable	Unavailable	Unavailable	Unavailable		Unavailable	Unavailable	Unavailable	Unavailable	Unavailable
Serbia	16.35	Private	Unavailable	Unavailable	Unavailable	Unavailable		Unavailable	Public	Available	Bupropion	150 mg (30 tablets)	23.42	1.43	Unaffordable	Available	Bupropion	150 mg (NIA)	NIA
Somalia	5.05	Private	Unavailable	Unavailable	Unavailable	Unavailable		Unavailable	Private	Unavailable	Unavailable	Unavailable	Unavailable		Unavailable	NIA	NIA	NIA	NIA
South Africa	9.37	Private	Unavailable	Unavailable	Unavailable	Unavailable		Unavailable	Public	Unavailable	Unavailable	Unavailable	Unavailable		Unavailable	Unavailable	Unavailable	Unavailable	Unavailable
South Sudan	5.05	Private	Unavailable	Unavailable	Unavailable	Unavailable		Unavailable	No information	NIA	NIA	NIA	NIA		NIA	NIA	NIA	NIA	NIA
Sri Lanka	1.35	Private	Unavailable	Unavailable	Unavailable	Unavailable		Unavailable	Public	Unavailable	Unavailable	Unavailable	Unavailable		Unavailable	Unavailable	Unavailable	Unavailable	Unavailable
Sudan	0.03	Private	Unavailable	Unavailable	Unavailable	Unavailable		Unavailable	Public	Unavailable	Unavailable	Unavailable	Unavailable		Unavailable	Unavailable	Unavailable	Unavailable	Unavailable
Syria	0.15	Private	Unavailable	Unavailable	Unavailable	Unavailable		Unavailable	Public	Unavailable	Unavailable	Unavailable	Unavailable		Unavailable	Unavailable	Unavailable	Unavailable	Unavailable
Tanzania	2.18	Private	Unavailable	Unavailable	Unavailable	Unavailable		Unavailable	Public	Unavailable	Unavailable	Unavailable	Unavailable		Unavailable	Unavailable	Unavailable	Unavailable	Unavailable
Thailand	9.05	Public	Unavailable	Unavailable	Unavailable	Unavailable		Unavailable	Public	Unavailable	Unavailable	Unavailable	Unavailable		Unavailable	NIA	NIA	NIA	NIA
The Gambia	0.91	Private	Unavailable	Unavailable	Unavailable	Unavailable		Unavailable	Public	Unavailable	Unavailable	Unavailable	Unavailable		Unavailable	Unavailable	Unavailable	Unavailable	Unavailable

Timor Leste	4.43	No information	NIA	NIA	NIA	NIA		NIA	Private	Unavailable	Unavailable	Unavailable	Unavailable		Unavailable	NIA	NIA	NIA	NIA
Tunisia	4.74	Public	Unavailable	Unavailable	Unavailable	Unavailable		Unavailable	Public	Unavailable	Unavailable	Unavailable	Unavailable		Unavailable	Unavailable	Unavailable	Unavailable	Unavailable
Turkiye	13.90	Private	Unavailable	Unavailable	Unavailable	Unavailable		Unavailable	Private	Unavailable	Unavailable	Unavailable	Unavailable		Unavailable	Unavailable	Unavailable	Unavailable	Unavailable
Uganda	0.07	Private	Unavailable	Unavailable	Unavailable	Unavailable		Unavailable	NGO	Unavailable	Unavailable	Unavailable	Unavailable		Unavailable	Unavailable	Unavailable	Unavailable	Unavailable
Venezuela	0.94	Private	Unavailable	Unavailable	Unavailable	Unavailable		Unavailable	Private	Unavailable	Unavailable	Unavailable	Unavailable		Unavailable	NIA	NIA	NIA	NIA
Viet Nam	6.00	Private	Unavailable	Unavailable	Unavailable	Unavailable		Unavailable	Public	Unavailable	Unavailable	Unavailable	Unavailable		Unavailable	Unavailable	Unavailable	Unavailable	Unavailable
Yemen	3.23	Public	Unavailable	Unavailable	Unavailable	Unavailable		Unavailable	Private	Unavailable	Unavailable	Unavailable	Unavailable		Unavailable	NIA	NIA	NIA	NIA
Zambia	2.59	Private	Unavailable	Unavailable	Unavailable	Unavailable		Unavailable	Public	Unavailable	Unavailable	Unavailable	Unavailable		Unavailable	Unavailable	Unavailable	Unavailable	Unavailable
Zimbabwe	11.58	Private	Unavailable	Unavailable	Unavailable	Unavailable		Unavailable	Public	Unavailable	Unavailable	Unavailable	Unavailable		Unavailable	Unavailable	Unavailable	Unavailable	Unavailable

Supplementary table 8: Availability of medications for smoking cessation by WHO regions and income level in pharmacies, HCFs and CMSs

Healthcare facility (HCF); Central medical Store (CMS)

			Nicotine gums			Nicotine transdermal patches			Bupropion tablets		
**Country**	**Income group**	**WHO Region**	**pharmacy**	**HCF**	**CMS**	**pharmacy**	**HCF**	**CMS**	**pharmacy**	**HCF**	**CMS**
Albania	Upper middle income	Europe	Unavailable	Unavailable	NIA	Unavailable	Unavailable	NIA	Unavailable	Unavailable	NIA
Algeria	Lower middle income	Africa	Unavailable	Unavailable	Unavailable	Unavailable	Unavailable	Unavailable	Unavailable	Unavailable	Unavailable
Angola	Lower middle income	Africa	Unavailable	Unavailable	Unavailable	Unavailable	Unavailable	Unavailable	Unavailable	Unavailable	Unavailable
Argentina	Upper middle	Americas	Available	Unavailable	Unavailable	Unavailable	Unavailable	Unavailable	Unavailable	Unavailable	Unavailable

	income										
Bosnia and Herzegovina	Upper middle income	Europe	Available	Available	NIA	Available	Available	NIA	Unavailable	Unavailable	NIA
Brazil	Upper middle income	Americas	Available	Unavailable	Unavailable	Available	Unavailable	Unavailable	Unavailable	Unavailable	Unavailable
Burkina Faso	Low income	Africa	Available	Unavailable	NIA	Unavailable	Unavailable	NIA	Unavailable	Unavailable	NIA
Cameroon	Lower middle income	Africa	Available	Unavailable	Unavailable	Unavailable	Unavailable	Unavailable	Unavailable	Unavailable	Unavailable
Chad	Low income	Africa	NIA	NIA	Unavailable	NIA	NIA	Unavailable	NIA	NIA	Unavailable
China	Upper middle income	Western Pacific	Available	Unavailable	Unavailable	Available	Unavailable	Unavailable	Unavailable	Unavailable	Unavailable
Democratic Republic of Congo	Low income	Africa	Unavailable	Unavailable	Unavailable	Unavailable	Unavailable	Unavailable	Unavailable	Unavailable	Unavailable
Ecuador	Upper middle income	Americas	Unavailable	Unavailable	Unavailable	Unavailable	Unavailable	Unavailable	Unavailable	Unavailable	Unavailable
Egypt	Lower middle income	Eastern Mediterranean	Available	Unavailable	Unavailable	Available	Unavailable	Unavailable	Unavailable	Unavailable	Unavailable
Eswatini	Lower middle income	Africa	Unavailable	NIA	Unavailable	Unavailable	NIA	Unavailable	Unavailable	NIA	Unavailable

Ethiopia	Low income	Africa	Unavailable	Unavailable	Unavailable	Unavailable	Unavailable	Unavailable	Unavailable	Unavailable	Unavailable
Ghana	Lower middle income	Africa	Unavailable	Unavailable	Unavailable	Unavailable	Unavailable	Unavailable	Unavailable	Unavailable	Unavailable
Guinea	Low income	Africa	Unavailable	NIA	Unavailable	Unavailable	NIA	Unavailable	Unavailable	NIA	Unavailable
Honduras	Lower middle income	Americas	Unavailable	Unavailable	NIA	Unavailable	Unavailable	NIA	Unavailable	Unavailable	NIA
India	Lower middle income	South-East Asia	Available	Unavailable	Unavailable	Unavailable	Unavailable	Unavailable	Unavailable	Unavailable	Unavailable
Indonesia	Lower middle income	South-East Asia	Unavailable	Unavailable	Unavailable	Unavailable	Unavailable	Unavailable	Unavailable	Unavailable	Unavailable
Iran	Lower middle income	Eastern Mediterranean	Available	Unavailable	Available	Unavailable	Unavailable	Unavailable	Unavailable	Unavailable	Unavailable
Iraq	Upper middle income	Eastern Mediterranean	Unavailable	Unavailable	NIA	Unavailable	Unavailable	NIA	Unavailable	Unavailable	NIA
Kenya	Lower middle income	Africa	Unavailable	Unavailable	NIA	Unavailable	Available	NIA	Unavailable	Unavailable	NIA
Kyrgyzstan	Lower middle income	Europe	Unavailable	Unavailable	Unavailable	Unavailable	Available	Available	Unavailable	Unavailable	Unavailable
Lesotho	Lower middle	Africa	NIA	Available	Unavailable	NIA	Available	Unavailable	NIA	Unavailable	Unavailable

	income										
Libya	Upper middle income	Eastern Mediterranean	Available	Unavailable	Unavailable	Unavailable	Unavailable	Unavailable	Unavailable	Unavailable	Unavailable
Malawi	Low income	Africa	Unavailable	Unavailable	Unavailable	Unavailable	Unavailable	Unavailable	Unavailable	Unavailable	Unavailable
Malaysia	Upper middle income	Western Pacific	Available	Unavailable	Unavailable	Available	Unavailable	Unavailable	Unavailable	Unavailable	Unavailable
Mali	Low income	Africa	Available	Unavailable	Unavailable	Unavailable	Unavailable	Unavailable	Unavailable	Unavailable	Unavailable
Mexico	Upper middle income	Americas	Unavailable	Unavailable	NIA	Available	Unavailable	NIA	Unavailable	Unavailable	NIA
Moçambique	Low income	Africa	Unavailable	Unavailable	Unavailable	Unavailable	Unavailable	Unavailable	Unavailable	Unavailable	Unavailable
Mongolia	Lower middle income	Western Pacific	Unavailable	Unavailable	Unavailable	Available	Unavailable	Available	Unavailable	Unavailable	Unavailable
Nepal	Lower middle income	South-East Asia	Unavailable	Unavailable	Unavailable	Unavailable	Unavailable	Unavailable	Unavailable	Unavailable	Unavailable
Niger	Low income	Africa	Available	Unavailable	Unavailable	Available	Unavailable	Unavailable	Unavailable	Unavailable	Unavailable
Nigeria	Lower middle income	Africa	Available	Unavailable	Unavailable	Unavailable	Unavailable	Unavailable	Available	Unavailable	Unavailable
North Macedonia	Upper middle income	Europe	Unavailable	Unavailable	Unavailable	Unavailable	Unavailable	Unavailable	Unavailable	Unavailable	Unavailable

Pakistan	Lower middle income	Eastern Mediterranean	Unavailable	Unavailable	Unavailable	Unavailable	Unavailable	Unavailable	Unavailable	Unavailable	Unavailable
Peru	Upper middle income	Americas	Unavailable	Unavailable	Unavailable	Unavailable	Unavailable	Unavailable	Unavailable	Unavailable	Unavailable
Romania	Upper middle income	Europe	Available	Available	Available	Available	Unavailable	Available	Available	Available	Available
Rwanda	Low income	Africa	Unavailable	Unavailable	NIA	Unavailable	Unavailable	NIA	Unavailable	Unavailable	NIA
Senegal	Lower middle income	Africa	Unavailable	Unavailable	Unavailable	Unavailable	Unavailable	Unavailable	Unavailable	Unavailable	Unavailable
Serbia	Upper middle income	Europe	Available	Available	Available	Unavailable	Available	Available	Unavailable	Available	Available
Somalia	Low income	Eastern Mediterranean	Unavailable	Unavailable	NIA	Unavailable	Unavailable	NIA	Unavailable	Unavailable	NIA
South Africa	Upper middle income	Africa	Available	Unavailable	Unavailable	Available	Unavailable	Unavailable	Unavailable	Unavailable	Unavailable
South Sudan	Low income	Africa	Unavailable	NIA	NIA	Unavailable	NIA	NIA	Unavailable	NIA	NIA
Sri Lanka	Lower middle income	South-East Asia	Unavailable	Unavailable	Unavailable	Unavailable	Unavailable	Unavailable	Unavailable	Unavailable	Unavailable
Sudan	Low income	Eastern Mediterran	Unavaila	Unavailable	Unavailable	Unavailable	Unavailable	Unavailable	Unavailable	Unavailable	Unavailable

		ean	ble								
Syria	Low income	Eastern Mediterranean	Unavailable	Unavailable	Unavailable	Unavailable	Unavailable	Unavailable	Unavailable	Unavailable	Unavailable
Tanzania	Lower middle income	Africa	Unavailable	Unavailable	Unavailable	Unavailable	Unavailable	Unavailable	Unavailable	Unavailable	Unavailable
Thailand	Upper middle income	South-East Asia	Unavailable	Unavailable	NIA	Unavailable	Unavailable	NIA	Unavailable	Unavailable	NIA
The Gambia	Low income	Africa	Available	Unavailable	Unavailable	Available	Unavailable	Unavailable	Unavailable	Unavailable	Unavailable
Timor Leste	Lower middle income	South-East Asia	NIA	Unavailable	NIA	NIA	Unavailable	NIA	NIA	Unavailable	NIA
Tunisia	Lower middle income	Eastern Mediterranean	Unavailable	Unavailable	Unavailable	Unavailable	Unavailable	Unavailable	Unavailable	Unavailable	Unavailable
Turkiye	Upper middle income	Europe	Available	Available	Available	Available	Available	Available	Unavailable	Unavailable	Unavailable
Uganda	Low income	Africa	Unavailable	Unavailable	Unavailable	Unavailable	Unavailable	Unavailable	Unavailable	Unavailable	Unavailable
Venezuela		Americas	Unavailable	Unavailable	NIA	Unavailable	Unavailable	NIA	Unavailable	Unavailable	NIA
Viet Nam	Lower middle income	Western Pacific	Unavailable	Unavailable	Unavailable	Unavailable	Unavailable	Unavailable	Unavailable	Unavailable	Unavailable

Yemen	Low income	Eastern Mediterranean	Unavailable	Unavailable	NIA	Unavailable	Unavailable	NIA	Unavailable	Unavailable	NIA
Zambia	Low income	Africa	Available	Unavailable	Unavailable	Available	Unavailable	Unavailable	Unavailable	Unavailable	Unavailable
Zimbabwe	Lower middle income	Africa	Unavailable	Unavailable	Unavailable	Unavailable	Unavailable	Unavailable	Unavailable	Unavailable	Unavailable

Supplementary Table 9: Cost of cigarettes in LMIC: One months cost based on average consumption of 10 cigarettes/day [14]

Country	Cost of 20 cigarettes ($)	Cost of 10 cigarettes ($)	Cost of one month of cigarettes (10 cigarettes/day for 30 days) ($)
Albania	1.80	0.9	27
Algeria	1.05	0.53	15.75
Angola	0.99	0.50	14.88
Argentina	0.21	0.10	3.09
Bosnia and Herzegovina	2.55	1.28	38.29
Brazil	0.95	0.48	14.25
Burkina Faso	0.96	0.48	14.4
Cameroon	0.80	0.4	12
Chad	0.80	0.4	12
China	0.43	0.21	6.43
Democratic Republic of Congo	0.50	0.25	7.5
Ecuador	5.10	2.55	76.5
Egypt	0.84	0.42	12.55
Eswatini	2.44	1.22	36.57
Ethiopia	0.48	0.24	7.13
Ghana	0.18	0.09	2.63
Guinea	0.46	0.23	6.91
Honduras	1.01	0.51	15.18
India	1.56	0.78	23.4
Indonesia	0.93	0.47	14.0
Iran	1.00	0.5	15
Iraq	0.35	0.18	5.25

Kenya	1.06	0.53	15.93
Kyrgyzstan	0.58	0.29	8.7
Lesotho	2.67	1.34	40.12
Libya	0.41	0.21	6.21
Malawi	0.20	0.1	3
Malaysia	2.68	1.34	40.14
Mali	0.40	0.2	6
Mexico	2.75	1.38	41.25
Moçambique	0.31	0.16	4.71
Mongolia	0.66	0.33	9.9
Nepal	0.40	0.2	5.93
Niger	1.00	0.5	15
Nigeria	0.48	0.24	7.2
North Macedonia	1.46	0.73	21.93
Pakistan	0.35	0.18	5.28
Peru	2.76	1.38	41.42
Romania	3.79	1.89	56.82
Rwanda	0.73	0.36	10.92
Senegal	1.11	0.55	16.59
Serbia	1.98	0.99	29.7
Somalia	0.49	0.25	7.41
South Africa	1.54	0.77	23.10
South Sudan	0.80	0.4	12
Sri Lanka	1.12	0.56	16.8
Sudan	0.06	0.03	0.9
Syria	0.10	0.05	1.5
Tanzania	0.80	0.4	12

Thailand	1.49	0.74	22.28
The Gambia	0.91	0.46	13.65
Timor Leste	2.00	1	30
Tunisia	0.54	0.27	8.06
Turkiye	0.61	0.31	9.21
Uganda	0.75	0.38	11.25
Venezuela	0.67	0.34	10.09
Viet Nam	0.27	0.13	4.02
Yemen	1.40	0.7	20.98
Zambia	1.36	0.68	20.35
Zimbabwe	0.16	0.08	2.35

Varenicline and Bupropion tablets limitations

Following completion of data collection it was discovered that varenicline was globally withdrawn from the market in 2021 by Pfizer [15], therefore making it widely unavailable. It remains on the latest WHO EML 2023 [16]. For this reason Varenicline availability was not explored in this study. Furthermore during the time of data collection, Glaxosmithkline (GSK) halted production of Bupropion brand “Zyban” on 1/12/2022 [17]. However some countries had already completed data submission, and others partially (Table 10). This may have affected the availability of Bupropion from countries that submitted data after this time. Bupropion has been reintroduced from October 2023, which was after completion of data collection. [17]

Table 10: Countries completion of data sets before and after Bupropion withdrawal from the market (1/12/2022)

	Before 01. December 2022	At study completion
Countries submitted any data	52	60
Countries submitted full datasets (1 pharmacy, 1 HCF, 1 CMS)	29	42
Countries that completed data submission with incomplete datasets (i.e. fewer than 3 facilities)	8	18

<u>**References**</u>

1. Stolbrink M, Ozoh OB, Halpin DMG Chronic Respiratory Diseases Medicines Survey Investigators Collaboration*, et al*Availability, cost and affordability of essential medicines for chronic respiratory diseases in low-income and middle-income countries: a cross-sectional study*Thorax* 2024;**79:**676-679.

2. Elm E von, Altman DG, Egger M, *et al.* The Strengthening the Reporting of Observational Studies in Epidemiology (STROBE) statement: guidelines for reporting observational studies. https://core.ac.uk/reader/33050540?utm_source=linkout (accessed Aug 23, 2023).

3. KoBoToolbox. 2020; published online Nov 2. https://www.kobotoolbox.org/ (accessed Nov 2, 2020)

4. WHO, Model List of Essential Medicines, 22nd list, 2021 24.5 Medications for disorders due to psychoactive substance use, https://www.who.int/publications/i/item/9789240077164
5. World Health Organisation, Global Health Observatory data repository: Retail price for a pack of 20 cigarettes Data by country (2022) https://apps.who.int/gho/data/view.main.TOBRETAILv accessed september 2023
6. World Bank. World Bank Country and Lending Groups. 2023; published online July 11. https://datahelpdesk.worldbank.org/knowledgebase/articles/906519-world-bank-country-and-lending-groups (accessed, 7/10/2023).

7. Exchange rates. Exchange rates. https://www.exchangerates.org.uk (accessed April 28, 2023)

8. Theilmann M, Lemp J M, Winkler V, Manne-Goehler J, Marcus M E, Probst C et al. Patterns of tobacco use in low and middle income countries by tobacco product and sociodemographic characteristics: nationally representative survey data from 82 countries *BMJ* 2022; 378 :e067582 doi:10.1136/bmj-2021-067582

9. National Institute for Clinical Excellence, British National Formulary, Bupropion Hydrochloride (2023): https://bnf.nice.org.uk/drugs/bupropion-hydrochloride/#indications-and-dose accessed 5/10/2023

10. National Institute for Clinical Excellence, British National formulary, Varenicline (2023) https://bnf.nice.org.uk/drugs/varenicline/ accessed 5/10/2023

11. International Labour Organization. International Labour Organization - Statistics on Wages. Geneva, Switzerland: United Nations, 2023 https://ilostat.ilo.org/topics/wages/.7/10/2023

12. WHO. Health Action International . 2nd edn. World Health Organization; Geneva: 2008. Measuring medicine prices, availability, affordability and price components

13. Stolbrink M, Thomson H, Hadfield RM, Ozoh OB, Nantanda R, Jayasooriya S, Allwood B, Halpin DMG, Salvi S, de Oca MM, Mortimer K, Rylance S. The availability, cost, and affordability of essential medicines for asthma and COPD in low-income and middle-income countries: a systematic review. Lancet Glob Health. 2022 Oct;10(10):e1423-e1442. doi: 10.1016/S2214-109X(22)00330-8. PMID: 36113528; PMCID: PMC9638033.

14. World Health Organisation, Global Health Observatory data repository: Retail price for a pack of 20 cigarettes Data by country (2022) https://apps.who.int/gho/data/view.main.TOBRETAILv

15. Lang AE, Berlin I, The Lancet, Unavailability of Varenicline: a global tragedy against the tobacco epidemic (published 12th May 2023) https://www.thelancet.com/action/showPdf?pii=S2213-2600%2823%2900184-4 accessed 18/10/2023

1. WHO, Model list of essential medicines, 23rd list, 2023: 24.5.2 Medicines for nicotine use disorders: https://iris.who.int/bitstream/handle/10665/371090/WHO-MHP-HPS-EML-2023.02-eng.pdf?sequence=1 Accessed 7/10/24

16. National Centre for Smoking Cessation and Training (NCSCT), Bupropion (Zyban) back in stock (2023): https://www.ncsct.co.uk/publications/zyban_stock#:~:text=On%201st%20December%202022%20GSK%20placed%20an%20immediate Accessed 7/10/24
